# Pooled analysis of recent studies on magnetic fields and childhood leukaemia

**DOI:** 10.1038/sj.bjc.6605838

**Published:** 2010-09-28

**Authors:** L Kheifets, A Ahlbom, C M Crespi, G Draper, J Hagihara, R M Lowenthal, G Mezei, S Oksuzyan, J Schüz, J Swanson, A Tittarelli, M Vinceti, V Wunsch Filho

**Affiliations:** 1 Department of Epidemiology, UCLA School of Public Health, 650 Charles Young Drive, Los Angeles, CA 90095, USA; 2 Division of Epidemiology, Institute of Environmental Medicine, Nobels väg 13, Karolinska Institutet, Stockholm SE-171 77, Sweden; 3 Department of Biostatistics, UCLA School of Public Health, Charles Young Drive, Los Angeles, CA 90095, USA; 4 Childhood Cancer Research Group, University of Oxford, Richards Building, Old Road Campus, Oxford OX3 7LG, UK; 5 Miyagi University, 1-1 Gakuen, Taiwa-cho Kurokawa-gun, Miyagi 981-3298, Japan; 6 School of Medicine and Menzies Research Institute, University of Tasmania, 17 Liverpool Street, Hobart, Tasmania 7000, Australia; 7 Environment Department, Electric Power Research Institute, 3420 Hillview Avenue, Palo Alto, CA 94304, USA; 8 Institute of Cancer Epidemiology, Danish Cancer Society, Strandboulevarden 49, Copenhagen DK-2100, Denmark; 9 National Grid, plc, 1-3 Strand, London WC2N 5EH, UK; 10 Cancer Registry and Environmental Epidemiology Unit, National Cancer Institute, Via Venezian, Milan 120133, Italy; 11 Department of Public Health Sciences, University of Modena and Reggio Emilia, Via Campi 287, Modena 41100, Italy; 12 School of Public Health, Av. Dr Arnaldo, 715, Universidade de São Paulo, 01246-904 São Paulo, Brazil

**Keywords:** magnetic fields, childhood leukaemia, pooled analysis, meta-analysis

## Abstract

**Background:**

Previous pooled analyses have reported an association between magnetic fields and childhood leukaemia. We present a pooled analysis based on primary data from studies on residential magnetic fields and childhood leukaemia published after 2000.

**Methods:**

Seven studies with a total of 10 865 cases and 12 853 controls were included. The main analysis focused on 24-h magnetic field measurements or calculated fields in residences.

**Results:**

In the combined results, risk increased with increase in exposure, but the estimates were imprecise. The odds ratios for exposure categories of 0.1–0.2 *μ*T, 0.2–0.3 *μ*T and ⩾0.3 *μ*T, compared with <0.1 *μ*T, were 1.07 (95% CI 0.81–1.41), 1.16 (0.69–1.93) and 1.44 (0.88–2.36), respectively. Without the most influential study from Brazil, the odds ratios increased somewhat. An increasing trend was also suggested by a nonparametric analysis conducted using a generalised additive model.

**Conclusions:**

Our results are in line with previous pooled analyses showing an association between magnetic fields and childhood leukaemia. Overall, the association is weaker in the most recently conducted studies, but these studies are small and lack methodological improvements needed to resolve the apparent association. We conclude that recent studies on magnetic fields and childhood leukaemia do not alter the previous assessment that magnetic fields are possibly carcinogenic.

Over the past three decades, potential health effects of residential and occupational exposure to extremely low-frequency electric and magnetic fields have been extensively investigated in epidemiological studies. Most attention has focused on a potential association between residential magnetic field exposure and childhood leukaemia. Almost all individual studies on magnetic fields and childhood leukaemia have found increased risks associated with the top percentiles of exposure levels; most of them, however, have involved a small number of exposed cases at the top percentiles. This has given rise to various interpretations. Two pooled analyses by Ahlbom *et al*, (2000) and Greenland *et al*, (2000), based on 9 and 12 studies, respectively, published up to 1999, have provided a basis for concluding that a consistent epidemiological association exists between residential exposure to magnetic fields and the risk of childhood leukaemia. Similar results were obtained by pooling data from four studies that included 24/48 h measurements, for exposure over the entire day and at night only (Schuz *et al*, 2007). Although hundreds of laboratory studies have been published, with a few reporting positive findings, most of the laboratory work has been negative. This has led to the general conclusion that robust, reliable and reproducible evidence of effects of magnetic fields at environmental levels on biological systems, either *in vivo* or *in vitro*, is lacking (IARC, 2002; WHO EHC, 2007). Thus, largely on the basis of epidemiological association of residential magnetic field exposure and childhood leukaemia, the International Agency for Research on Cancer has classified extremely low-frequency magnetic field exposure as being possibly carcinogenic to humans (Group 2B; IARC, 2002).

Since carrying out the pooled analyses, several new epidemiological studies have been published. The World Health Organization (WHO) reviewed results of the studies available through to 2006 in an Environmental Health Criteria (EHC) monograph (WHO EHC, 2007), with the conclusion that the ‘possibly carcinogenic’ classification does not change with the addition of new studies, but that the pooled analyses should be updated with the results from recent studies. In fact, such an analysis is identified as a high research priority in the WHO research agenda issued in 2007 (WHO, 2007).

We present a pooled analysis based on primary data of seven recent studies on magnetic fields and childhood leukaemia, to assess whether the combined results, adjusted for potential confounding, confirm the results of previous pooled analyses and whether there is an association between EMF exposure and childhood leukaemia.

## Materials and Methods

### Selection

We searched the published literature through PubMed, as well as references of identified papers, and conducted an informal survey of epidemiologists involved in magnetic field research to identify relevant recent and ongoing studies on residential magnetic field exposure and childhood leukaemia published since the previous pooled analyses of childhood leukaemia published in (Ahlbom *et al*, 2000; Greenland *et al*, 2000). To be included, studies had to provide data for children, provide data separately for leukaemia, be population based and provide measured or calculated residential magnetic fields inside a home. Studies that used distance to power lines as an exposure metric were also included, but not in the main analysis.

We identified 14 studies, of which seven met our inclusion criteria (Table 1). Appendix 1 summarises the methods and findings of studies that were not included. One study (Hoffmann *et al*, 2008) did not publish data on children and had a large overlap with a large countrywide German study (Schuz et al, 2001); to maintain independence of observations, only the countrywide German study (former West Germany) was included. Three studies were excluded because they were hospital based (Perez *et al*, 2005; Feizi and Arabi, 2007; Abdul Rahman *et al*, 2008). One study was excluded because it was a case-only study (Yang *et al*, 2008). Another study was excluded because it was exclusively of children with Down's syndrome, who are at substantially higher risk for leukaemia (Mejia-Arangure *et al*, 2007). One study, the Northern California Childhood Leukemia Study (NCCLS), was not made available in time for inclusion. However, the exposure assessment methods of this study were substantially different from all other measurement studies: a 30-min measurement was taken in the room with the median spot measurement after a survey of the entire residence, as compared with a 24-h or more measurement in the child's bedroom in all other measured field studies (Does *et al*, 2009). We attempted to obtain unpublished data from all known sources, and identified three additional studies that are underway, but with completion dates several years away.

### Materials

One of the included studies (Brazil) has not yet been published (Wunsch Filho, personal communications, 2009). All included studies used a matched case–control design, although the matching variables were not the same in all studies (Bianchi *et al*, 2000; Schuz *et al*, 2001; Kabuto *et al*, 2006; Lowenthal *et al*, 2007; Kroll *et al*, 2010; Malagoli *et al*, 2010). In the original publication of one of the Italian studies, some of the controls were selected nonconcurrently (Bianchi *et al*, 2000). For this publication, the time period for that study was extended by 5 years by adding new cases and controls and was limited to the period for which concurrent control selection was possible (1978–1997). As we wanted to use as many cases and controls as possible to increase the flexibility of the analysis (and for other methodological reasons as described in Greenland *et al*, 2000), we ignored the matching and instead included adjustment for age of diagnosis, sex and study. To make the data as consistent as possible across studies, we limited the age of diagnosis to 0–15 years inclusive and converted all measured and calculated field from milligauss to microtesla. However, it should be noted that the Brazilian study included children of age 8 years or younger only, because computerised records of birth certificates used for control selection were available only from 2000 onwards. It is also the only study that includes only acute lymphoblastic leukaemia (ALL) cases.

We focused on surrogates of magnetic fields at home. All studies had long-term measurements (Brazil, Germany, Japan) or calculated magnetic fields (Italy1, Italy2, UK), except for the Tasmanian study, which included only distance to power lines. The long-term measurement studies used metres placed in the child's bedroom. Long-term measurements were taken for 24 h in two studies (Brazil, Germany), and for a 1-week period in one study (Japan). Long-term measurements can be affected by short-duration exposure to high fields, e.g., from domestic electrical appliances, which are not part of the background field at home. We followed Ahlbom *et al* (2000) and used geometric means of the long-term measurements in our analyses to reduce such effects. Three studies (UK, Italy1, Italy2) provided calculated fields, on the basis of distance between the subject's home and the closest line, taking into account historical load conditions and other line characteristics.

The studies provided exposure measurements for home at diagnosis (Brazil, Italy1, Japan), for birth home (UK) or for the home in which the child lived for the longest period of time before diagnosis (Germany). Two studies (Italy2, Tasmania) evaluated multiple residences. Some mechanisms of carcinogenesis could operate perinatally or antenatally, others later in life. In the absence of a known mechanism for magnetic fields, there is little basis for preferring one period over another, and the choice in individual studies has been highly influenced by practicalities of study design. To select an exposure proxy for subjects from these studies, we used the diagnosis home if available; if not, we used the home in which the subject lived the longest, and if that is not available, we used birth home, on the basis that, for measurement studies, more recent measurements are probably more reliable.

A number of potential confounders such as the type of dwelling, mobility, urbanisation, socioeconomic status (SES) and traffic exhaust were available in some studies (see Table 1). The number, type and coding of potential confounders differed among the studies. We examined mobility (dichotomised as one or more than one residence before diagnosis) and SES. Variables coding SES differed by study. We standardised SES to a three-level ordinal variable (low, medium and high) on the basis of SES in each country. Other potential confounders were available from too few studies to merit examination.

### Statistical methods

The analysis plan largely followed that of the pooled analysis of Ahlbom *et al* (2000). An analysis using exposure as a linear predictor was conducted for a likelihood ratio test of homogeneity of effects across studies. In most analyses, increasing exposure categories of 0.1–<0.2 *μ*T, 0.2–<0.3 *μ*T and ⩾0.3 *μ*T, with reference category <0.1 *μ*T, were used. A highest cutoff point of 0.3 *μ*T was chosen to obtain more stable results for the high-exposure category and to enable a direct comparison with results obtained by Greenland (Greenland *et al*, 2000). For comparison with results in Ahlbom, we also present some results with the highest cutoff point of 0.4 *μ*T. Data were analysed using both ordinary logistic regression, with fixed intercepts to adjust for study, and mixed effects logistic regression, with random intercepts and exposure effect coefficients for study. Ordinary and mixed effects logistic regression yielded similar results; hence, we present results of the ordinary logistic regression analysis only. We also obtained odds ratios using a moving window of exposure. These analyses used exposure categories of 0.1–<0.2, 0.15–<0.25, 0.20–<0.30, 0.25–<0.35, ⩾0.30, ⩾0.35 and ⩾0.40, with reference category <0.1 *μ*T, and were adjusted for age, sex and study. We estimated the trend in the log odds of being a case using a generalised additive model (GAM) (Hastie and Tibshirani, 1990) using a nonparametric curve (natural cubic smoothing spline with interior and boundary knots at the unique values of exposure) to estimate the risk associated with exposure, while controlling for study, age and sex. As a sensitivity analysis, we used a range of smoothing parameters (degrees of freedom, d.f.). These results were obtained using the gam package in R version 2.9.2 (R Development Core Team, 2009). Other analyses were conducted using Stata (StataCorp, 2007).

## Results

Of the included studies, four were conducted in Europe, and one each was conducted in Japan, Brazil and Australia. Table 1 shows the numbers of cases and controls for each study, along with variables supplied by those studies. There was a total of 10 865 cases and 12 853 controls with exposure surrogates; however, total numbers in the high-exposure categories were small, even for this large data set.

Table 2 presents the absolute numbers of subjects by case–control status, study and exposure level. The UK study provided by far the largest number of cases and controls, i.e., 89 and 75% however, influence on results is more dependent on the numbers in the high-exposure category, and thus Brazil with high numbers of exposed was expected to be the most influential. Overall, in the highest-exposure category (⩾0.3 *μ*T), there were 26 cases and 50 controls, 11 and 30 of them from the study in Brazil. Four studies (Germany, Italy1, Italy2 and Japan) provided histological type of leukaemia. Among subjects with data on type of leukaemia available, 86% were ALL cases. Numbers for other subtypes were too low to support additional analysis by subtype.

Table 3 summarises the main results. We present results for geometric means for long-term measurements (results for arithmetic means were similar) for each study adjusted for basic potential confounders, and separately for measured and calculated field studies, as well as combined results. A likelihood ratio test comparing models with and without random effects for exposure did not detect heterogeneity (*P*=0.201), supporting the pooling of studies.

In most individual studies and in the combined results, the risk increased with increase in exposure, although the estimates were imprecise. For calculated field studies, the number of subjects in high-exposure categories was often too small to provide reliable estimates. As Brazil was the most influential study in terms of the number of highly exposed subjects, and included only young and only ALL cases, we present results with and without Brazil. Influence analysis omitting one study at a time confirmed that Brazil was the most influential study (results not shown). Without Brazil, the summary odds ratio for ⩾0.3 *μ*T *vs* <0.1 *μ*T is 1.56 (95% CI 0.78–3.10), which is close to the age, sex and study-adjusted summary OR of 1.68 (95% CI 1.23–2.31) obtained in the pooled analysis of Greenland (Greenland *et al*, 2000), but less precise. In individual studies and in combined results, the number of observed cases ⩾0.3 *μ*T was higher than the expected number obtained by modelling the probability of membership in exposure categories on the basis of the distribution of controls, including covariates.

For a more direct comparison of the current pooled results with those of Ahlbom *et al*, we conducted an analysis using the same cutoff points. Our overall risk estimates, although compatible with previously reported estimates, are substantially lower (Table 4). This is particularly true for studies on measured fields, a result heavily influenced by the Brazilian study. The combined OR for ⩾0.4 *μ*T *vs* <0.1 *μ*T with Brazil omitted was 2.02 (95% CI 0.87–4.69), whereas combined ORs when omitting other single studies ranged from 1.32 to 1.49. When the Brazilian study is excluded from the analysis, our point estimates are very close to the results of Ahlbom *et al.* The same is true when a cutoff point ⩾0.3 *μ*T is used, rather than ⩾0.4 *μ*T.

**Figure 1:**
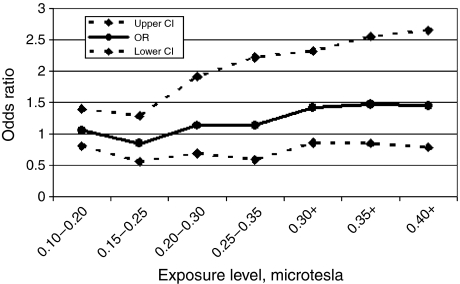
Odds ratios (95% CI) for moving window of exposure levels, adjusted for age, sex, SES and study. Reference level: <0.1 *μ*T.

Odds ratio estimates using categorical cutoff points and involving relatively small numbers of subjects are vulnerable to unstable results. To address this concern, we also calculated odds ratios using a moving window of exposure levels (Figure 1). These results also suggested a possible trend of increasing risk with increase in exposure; however, the estimates were imprecise.

**Figure 2:**
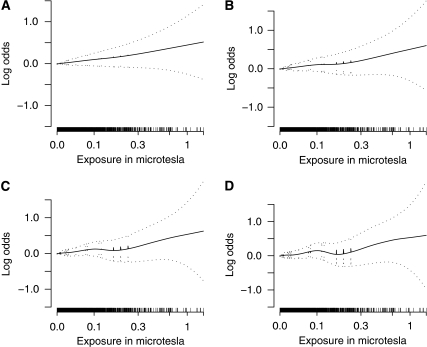
Nonparametric estimates of trend in log odds of being a case with a range of levels of smoothing (**A**. 2 d.f.; **B**. 3 d.f.; **C**. 4 d.f.; **D**. 5 d.f.) from a generalised additive model, with adjustment for study, age of diagnosis and sex. Outer dotted lines represent 95% confidence limits.

An ordinary logistic regression analysis using exposure as a continuous linear predictor yielded OR=1.11 (95% CI 0.98–1.26) for each increase of 0.2 *μ*T, adjusting for age and sex. However, we prefer using a GAM, which is a more flexible modelling approach that provides a nonparametric estimate of the association between exposure and risk while controlling for potential confounders. Figure 2 presents the GAM nonparametric estimate of the trend in the log odds of being a case, with adjustment for study, age and sex. As a sensitivity analysis, we present results for a range of smoothing parameters, expressed as d.f., with models with more d.f. reflecting more fidelity to the data and models with fewer d.f. yielding more smoothing. Confidence limits widen as exposure increases, reflecting smaller number of subjects at high exposure levels. Although the curve suggests a positive exposure–response relationship, the width of the confidence bands indicates that a variety of exposure–response relationships, including no increase in risk, are compatible with the data.

Table 5 presents sensitivity and subgroup analyses in which we examine whether results change with adjustments for potential confounders and to what extent results are limited to a particular subgroup. Not all potential confounders were available in all studies. Analyses adjusting for confounding were carried out on the subset of studies and subjects for which data on the confounder were available. Most adjustments did not make appreciable changes in odds ratio estimates. Risks were a little higher for ALL and for a younger age group, and a little lower for residences at birth, despite a suggestion from one study (Lowenthal *et al*, 2007) that exposure at birth might carry particular risks. Neither an adjustment for mobility nor restriction to subjects who lived in a single residence before diagnosis changed the risk estimates appreciably. All confidence intervals included the null value.

In very early studies on magnetic field exposure, distance from power lines was used as a proxy for magnetic fields, but distance alone is a poor predictor of magnetic fields when a study involves lines of varying characteristics, as highlighted in a recent methodological paper (Maslanyj *et al*, 2009). Draper *et al*, (2005) found elevated risks at distances well beyond the point at which the magnetic fields from power lines would be elevated, but were unable to offer an explanation for this finding. Using the pooled data, we, similar to Draper *et al* (2005), evaluated the risk of childhood leukaemia as it relates to distance as an ‘exposure’ in its own right and not as a substitute for magnetic fields.

The results for risk of childhood leukaemia as related to distance based on six studies (all except Germany) are shown in Table 6. Risk estimates increase with a decrease in distance, and the risk estimate for the closest band (⩽50 m) is the highest and relatively precise, but full exploration of how this effect occurs will require consideration of the different voltage lines involved and the effect of alternative reference levels.

## Discussion

We conducted a pooled analysis of seven recent epidemiological studies on the association between residential magnetic field exposure and childhood leukaemia. Pooled analysis, considered the gold standard for synthesising results from multiple studies, allows for comparison across different studies and metrics, free of artefacts introduced by analytical differences, and for derivation of statistically more stable results (Kheifets *et al*, 2006). Pooled analysis uses raw data from previous studies, and thus can apply identical analyses to all included studies. The choices of cutoff points, reference groups, metrics, etc., in a pooled analysis may differ from the choices made in the original studies and may result in changes in the study-specific effect estimates. Despite strengths, results from pooled analyses are prone to the same biases operating in the original studies. Studies using measurements generally have low participation rates, which might have led to selection bias (Mezei and Kheifets, 2006; Schuz and Ahlbom, 2008). Studies estimating calculated fields do not require participation and are thus less vulnerable to selection bias, but they neglect sources of magnetic fields other than high-voltage power lines and are thus likely to introduce exposure misclassification and loss of statistical power.

Our results, adjusting for potential confounding, broadly confirm the results of the previous pooled analyses by Greenland and Ahlbom, although the association is weaker when all studies are included. Our results are highly dependent on one study from Brazil that has greater influence because of comparatively high numbers of cases and controls at the upper exposure level. Possible explanations for the weaker association seen in the study from Brazil include: this study is affected by a bias that masks a true association more than other studies; this study is less affected by a bias evident in other studies that creates a spurious or stronger association; or that this is only a random variation.

Several unique features of the Brazilian study raise questions about the potential for bias. On one hand, it focuses on ALL, a more specific definition of disease, and on children <8 years of age, making it more likely that residential exposures are representative of total exposure. However, our subgroup analyses of ALL and of younger ages showed no strong indication that specificity of diagnosis or age is important. On the other hand, there are several limitations that might have led to bias. It is common in Brazil to move close to the treating hospital, and subjects who moved after diagnosis were not included, as it was logistically infeasible to conduct measurements in the homes in which they lived before diagnosis. In addition, participation between cases and controls was highly differential, in part because of the use of birth certificates as a source for controls and the difficulty in tracing individuals. As a result, 94.2% of controls in the Brazilian study have lived in a single residence, compared with 54.0% of cases. Thus, we speculate that the Brazilian study unduly pushes our risk estimates down. This is confirmed by an analysis of Brazilian data limited to residentially stable subjects: OR for ⩾0.3 *μ*T *vs* <0.1 *μ*T increases to 1.46 (95% CI 0.61–3.50, adjusted for age, sex and SES).

Although our results are compatible with no effect, when considering all studies combined, our findings suggest a small increase in risk with increasing exposure, regardless of the model chosen. Without the Brazilian study, our estimates are very close to those by Ahlbom *et al*, but less precise. Importantly, this pooled analysis, as compared with previous pooled analyses, includes a wider range of countries, including those in Asia and South America.

Most of the studies not included reported much higher estimates of risk, but had serious methodological problems. The addition of the one study that met our inclusion criteria but was not made available in time for this analysis, the Northern California Childhood Leukaemia Study, changes the risk estimates only slightly, resulting in OR=1.29 (0.81–2.06) for exposure ⩾0.3 *μ*T (results obtained using counts of cases and controls in exposure categories for NCCL, which were available from the conference presentation; results adjusted for study only, as confounders were not available). Recall, however, that the measurements in this study are substantially different in length and most importantly in the location chosen for measurements.

In conclusion, our results are in line with previous pooled analyses showing an association between residential magnetic field exposure and childhood leukaemia, but the association is weaker in recent studies and imprecise because of small numbers of highly exposed individuals. At the same time, recent studies are small and lack methodological improvements needed to resolve scientific uncertainties regarding the apparent association. In the IARC classification scheme, a key issue is whether ‘chance, bias and confounding could be ruled out with reasonable confidence’. Our results, added to the previous pooled analyses, make chance less likely, but do not rule out bias or confounding, as whatever bias or confounding was present in previous studies could be present in these studies as well. Therefore, our results support conclusions of the WHO EHC (WHO EHC, 2007) and the European Union Scientific Committee on Emerging and Newly Identified Health Risks (Scientific Committee on Emerging and Newly Identified Health Risks, 2007) that recent studies on magnetic fields and childhood leukaemia do not alter the previous assessment that magnetic fields are possibly carcinogenic to humans.

## Figures and Tables

**Table 1:** Characteristics of studies in the pooled analysis of childhood leukaemia and EMF exposure

	**Subjects**			**Exposure measurement**				**Potential confounders**						
**Study**	**Cases**	**Controls**	**Year of diagnosis**	**Home targeted for assessment**	**Long-term measurements**	**Calculated fields**	**Distance**	**Sex**	**Date of birth/age**	**Dwelling type**	**Mobility**	**Socioeconomic status**	**Urbanisation**	**Traffic exhaust**
Brazil	162	565	2001–2009	Diagnosis	√		√	√	√		√	√		
Germany	514	1301	1988–1994	Longest	√			√	√	√	√	√	√	
Italy1	119	476	1978–1997	Diagnosis		√	√	√	√			√		√
Italy2	46	184	1986–2007	Diagnosis		√	√	√	√		√	√		
Japan	312	603	1999–2001	Diagnosis	√		√	√	√		√	√	√	
Tasmania	47	47	1972–1980	Diagnosis			√	√	√		√	√		
UK	9695	9695	1962–1995	Birth		√	√	√	√			√	√	

Abbreviation: EMF=electronic and magnetic fields.

**Table 2:** Absolute numbers of childhood leukaemia cases and controls by study and exposure level

**Total leukaemia cases**						**Acute lymphoblastic leukaemia cases**				
	**<0.1 *μ*T**	**0.1–0.2 *μ*T**	**0.2–0.3 *μ*T**	**⩾0.3 *μ*T**	**Total**	**<0.1 *μ*T**	**0.1–0.2 *μ*T**	**0.2–0.3 *μ*T**	**⩾0.3 *μ*T**	**Total**
*Measurement studies*										
Brazil	120	23	8	11	162	120	23	8	11	162
Germany	474	30	6	4	514	418	24	6	4	452
Japan	279	17	8	8	312	225	13	6	7	251
Total	**873**	**70**	**22**	**23**	**988**	**763**	**60**	**20**	**22**	**865**
*Controls*										
Brazil	416	90	29	30	565					
Germany	1212	74	12	3	1301					
Japan	552	29	10	12	603					
Total	**2180**	**193**	**51**	**45**	**2469**					
										
*Calculated field studies*										
Italy1	116	3	0	0	119	93	2	0	0	95
Italy2	45	0	0	1	46	36	0	0	1	37
UK	9657	6	0	2	9665					n/a
Total	**9818**	**9**	**0**	**3**	**9830**					
*Controls*										
Italy1	469	5	1	1	476					
Italy2	181	1	0	2	184					
UK	9671	3	1	2	9677					
Total	**10 321**	**9**	**2**	**5**	**10 337**					

**Table 3:** Odds ratios (95% CI) for childhood leukaemia by exposure level with adjustment for age, sex and SES

**Type of study**	**0.1–<0.2 *μ*T**	**0.2–<0.3 *μ*T**	**⩾0.3 *μ*T**	**O^a^**	**E^a^**
*Measurement studies*					
Brazil	0.89 (0.54–1.47)	0.97 (0.43–2.19)	1.26 (0.61–2.62)	11	9.3
Germany	1.02 (0.66–1.59)	1.32 (0.49–3.54)	3.05 (0.68–13.8)	4	1.2
Japan	1.10 (0.59–2.04)	1.50 (0.58–3.88)	1.40 (0.56–3.49)	8	6.2
					
*Calculated field studies*					
Italy1	2.36 (0.55–10.1)	0 cases/1 controls	0 cases/1 controls	0	0.2
Italy2	0 cases/1 controls	0 cases/0 controls	2.26 (0.20–25.9)	1	0.4
UK	2.01 (0.50–8.03)	0 cases/1 controls	0.98 (0.14–6.97)	2	1.9
					
*Summary* b					
Measurement studies	1.00 (0.74–1.33)	1.19 (0.71–1.99)	1.49 (0.88–2.51)	23	16.6
Calculated field studies	2.02 (0.75–5.41)	0 cases/2 controls	1.15 (0.25–5.32)	3	2.7
All studies	1.07 (0.81–1.41)	1.16 (0.69–1.93)	1.44 (0.88–2.36)	26	18.9
All without Brazil	1.16 (0.83–1.61)	1.30 (0.67–2.54)	1.56 (0.78–3.10)	15	9.9

Abbreviations: CI=confidence interval; SES=socioeconomic status.

Reference level: <0.1 *μ*T.

a Observed (O) and expected (E) number of cases ⩾0.3 *μ*T, with expected numbers obtained by modelling probability of membership in exposure categories based on the distribution of controls including covariates.

b Adjusted for study, age, sex and SES.

**Table 4:** Comparison of summary odds ratios in current pooled analysis update with pooled analysis of Ahlbom *et al* (2000); adjusted for age, sex, SES and study

**Type of study**	**0.1–<0.2 *μ*T**	**0.2–<0.4 *μ*T**	**⩾0.4 *μ*T**
*Measurement studies*			
Ahlbom *et al*	1.05 (0.86–1.28)	1.15 (0.85–1.54)	1.87 (1.10–3.18)
Current update	1.00 (0.74–1.33)	1.29 (0.83–2.02)	1.41 (0.73–2.71)
Current update with Brazil	1.05 (0.73–1.50)	1.36 (0.75–2.48)	2.23 (0.83–5.99)
			
*Calculated field studies*			
Ahlbom *et al*	1.58 (0.77–3.25)	0.79 (0.27–2.28)	2.13 (0.93–4.88)
Current update	2.02 (0.75–5.41)	0 cases/3 controls	1.68 (0.34–8.38)
			
*All studies*			
Ahlbom *et al*	1.08 (0.89–1.31)	1.11 (0.84–1.47)	2.00 (1.27–3.13)
Current update	1.07 (0.81–1.41)	1.22 (0.78–1.89)	1.46 (0.80–2.68)
Current update without Brazil	1.15 (0.83–1.61)	1.20 (0.67–2.17)	2.02 (0.87–4.69)

Abbreviation: SES=socioeconomic status.

Reference level: <0.1 *μ*T.

**Table 5:** Summary odds ratios (95% CI) for leukaemia by exposure level with adjustments for study and other potential confounders and within subgroups

	**Studies included**	**0.1–<0.2 *μ*T**	**0.2–<0.3 *μ*T**	**⩾0.3 *μ*T**
*Adjustments for potential confounders*				
Adjusted for age, sex	All	1.06 (0.81–1.40)	1.16 (0.69–1.93)	1.43 (0.88–2.35)
Adjusted for age, sex, SES		1.07 (0.81–1.41)	1.16 (0.69–1.93)	1.44 (0.88–2.36)
Adjusted for age, sex, SES	All but Italy1 and UK	0.99 (0.74–1.32)	1.19 (0.71–1.99)	1.51 (0.91–2.52)
Adjusted for age, sex, SES, mobility		1.00 (0.75–1.34)	1.18 (0.70–1.98)	1.52 (0.91–2.54)
				
*Analyses of subgroups* a				
Single residence before diagnosis	All but Italy1 and UK	1.15 (0.80–1.66)	1.23 (0.61–2.48)	1.44 (0.73–2.87)
Acute lymphocytic leukemia	All but UK	0.97 (0.71–1.31)	1.19 (0.70–2.02)	1.56 (0.93–2.60)
Birth homes^b^	All	1.10 (0.80–1.52)	1.19 (0.64–2.19)	1.31 (0.74–2.35)
Age of diagnosis ⩽8 years	All	1.08 (0.80–1.46)	1.16 (0.67–2.00)	1.45 (0.86–2.46)
Age of diagnosis ⩽8 years	All but Brazil	1.21 (0.83–1.78)	1.34 (0.63–2.86)	1.63 (0.75–3.54)
Age of diagnosis >8 years	All but Brazil	1.01 (0.50–2.02)	1.21 (0.29–4.99)	1.30 (0.30–5.63)

Abbreviations: CI=confidence interval; SES=socioeconomic status.

Reference level: <0.1 *μ*T.

a Adjusted for study, age, sex, socioeconomic status (SES).

b Includes children who always lived at the same residence.

**Table 6:** Odds ratios (95% CIs) for childhood leukaemia and distance from nearest power line, adjusted for study, age, sex and SES

**>200 m**		**>100–200 m**		**>50–100 m**		**⩽50 m**	
**Cases/controls**	**OR**	**Cases/controls**	**OR (95% CI)**	**Cases/controls**	**OR (95% CI)**	**Cases/controls**	**OR (95% CI)**
10 153/11 231	1.0	88/146	1.20 (0.90, 1.59)	49/75	1.30 (0.89, 1.91)	35/51	1.59 (1.02, 2.50)

Abbreviations: CI=confidence interval; OR=odds ratio; SES=socioeconomic status.

Reference level: >200 m.

**Table 7:** 

**Region and author**	**Year of diagnosis**	**Disease diagnosis**	**No. of cases/controls**	**Exposure measurements**	**Ages**	**Results**	**Reason for exclusion**
China, Shanghai: Yang *et al* (2008)	2006–2007	Acute childhood leukaemia	123/NA	Transformers or power lines within 50 m	0–15	COR=4.39 (95% CI 1.42–13.54) for interaction of XRCC1 Ex9þ16A gene and a residency within 50 m of power line/transformer	Case-only study
Cuba: Perez *et al* (2005)	1996–2000	Acute childhood leukaemia	NA	SM in each room and outside	0–15	OR=1.2 (median exposure) OR=6.72 (for 0.5 *μ*T) OR=45.15 (for 1 *μ*T)	Methods unclear and problematic
Iran: Feizi and Arabi (2007)	1998–2004	Acute childhood leukaemia	60/59	Distance CF	0–15	Distance: OR=8.76, (95% CI 1.74–58.4) Calculated fields: OR=3.60, (95% CI 1.11–12.4)	Hospital based Problematic magnetic fields calculations
Mexico City: Mejia-Arangure *et al* (2007)	1995–2003	Acute childhood leukaemia	42/124	SM distance	0–16	OR=4.1 (95% CI 1.05–13) for ⩾0.6 *μ*T *vs* ⩽0.1 *μ*T)	Included only children with Down's syndrome
Malaysia: Abdul Rahman *et al* (2008)	2001–2007	Acute childhood leukaemia	128/128	Distance	0–14	OR=2.3 (95% CI 1.18–4.49) for ⩽200 m *vs* >200 m	Hospital based
Northern Germany: Hoffmann *et al* (2008)	1986–1998	Leukaemia and lymphoma	97/187	SM in a subset	0–75	NA	Large overlap with study by Schuz (2001), which is included in this pooled analysis
United States, Northern California: Does *et al* (2009)	2004–2007	Childhood leukaemia	245/269	SM WC	0–8	OR=0.57 (95% CI 0.3–1.93) for >0.3 *μ*T *vs* <0.1 *μ*T	No long-term measurements in child's bedroom Data not available in time

Abbreviations: CF=calculated fields; CI=confidence interval; COR=case-only odds ratio; NA= not applicable; OR=odds ratio; SM=spot measurements; WC=wire codes; XRCC1=X-ray repair complementing defective repair in Chinese hamster cells 1.
