# Pooled analysis of recent studies of magnetic fields and childhood leukaemia

**DOI:** 10.1038/sj.bjc.6606003

**Published:** 2011-01-04

**Authors:** L Kheifets, A Ahlbom, C M Crespi, G Draper, J Hagihara, R M Lowenthal, G Mezei, S Oksuzyan, J Schüz, J Swanson, A Tittarelli, M Vinceti, V Wunsch Filho

**Correction to**: *British Journal of Cancer* (2010) **103**, 1128–1135; doi:10.1038/sj.bjc.6605838

During the final correction of the above paper prior to its publication in late 2010, an error was introduced in Table 4. In the third line, under the subheading ‘Measurement studies’, the category should read ‘Current update without Brazil’. The correct Table is now shown, below.

The publishers apologise for this mistake.

## Figures and Tables

**Table 4:** Comparison of summary odds ratios in the current pooled analysis update with the pooled analysis of Ahlbom *et al* (2000), adjusted for age, sex, SES and study

**Type of study**	**0.1–<0.2 *μ*T**	**0.2–<0.4 *μ*T**	**⩾0.4 *μ*T**
*Measurement studies*			
Ahlbom *et al*	1.05 (0.86–1.28)	1.15 (0.85–1.54)	1.87 (1.10–3.18)
Current update	1.00 (0.74–1.33)	1.29 (0.83–2.02)	1.41 (0.73–2.71)
Current update without Brazil	1.05 (0.73–1.50)	1.36 (0.75–2.48)	2.23 (0.83–5.99)
			
*Calculated field studies*			
Ahlbom *et al*	1.58 (0.77–3.25)	0.79 (0.27–2.28)	2.13 (0.93–4.88)
Current update	2.02 (0.75–5.41)	0 cases/3 controls	1.68 (0.34–8.38)
			
*All studies*			
Ahlbom *et al*	1.08 (0.89–1.31)	1.11 (0.84–1.47)	2.00 (1.27–3.13)
Current update	1.07 (0.81–1.41)	1.22 (0.78–1.89)	1.46 (0.80–2.68)
Current update without Brazil	1.15 (0.83–1.61)	1.20 (0.67–2.17)	2.02 (0.87–4.69)

Abbreviation: SES=socioeconomic status.

Reference level: <0.1 *μ*T.

